# Nursing documentation of pressure ulcers in nursing homes: comparison of record content and patient examinations

**DOI:** 10.1002/nop2.47

**Published:** 2016-03-07

**Authors:** Ruth‐Linda Hansen, Mariann Fossum

**Affiliations:** ^1^ Department of Health and Nursing ScienceFaculty of Health and Sport SciencesUniversity of Agder/Centre for Caring ResearchGrimstad/Southern NorwayNorway; ^2^ Deakin UniversitySchool of Nursing and MidwiferyDeakin Alfred Health Nursing Research CenterAlfred HealthMelbourneVictoriaAustralia

**Keywords:** Electronic health records, nursing home, patient examination, pressure ulcer

## Abstract

**Aim:**

The aim of this study was to describe the accuracy and quality of nursing documentation of the prevalence, risk factors and prevention of pressure ulcers, and compare retrospective audits of nursing documentation with patient examinations conducted in nursing homes.

**Design:**

This study used a cross‐sectional descriptive design.

**Method:**

A retrospective audit of 155 patients' records and patient examinations using the European Pressure Ulcer Advisory Panel form and the Braden scale, conducted in January and February 2013.

**Results:**

The prevalence of pressure ulcers was 38 (26%) in the audit of the patient records and 33 (22%) in patient examinations. A total of 17 (45%) of the documented pressure ulcers were not graded. When comparing the patient examinations with the patient record contents, the patient records lacked information about pressure ulcers and preventive interventions.

## Introduction

Pressure ulcers (PUs) remain a serious health problem for older adult patients in nursing homes (Kwong *et al*. 2009, Demarré *et al*. 2012, Bååth *et al*. 2014), despite a widespread focus on the prevention of PUs (Fossum *et al*. 2011, Beeckman *et al*. 2013, Bååth *et al*. 2014). Improving risk assessment, planning and documentation is important to help prevent PUs in nursing homes (Moore & Cowman 2012). However, risk assessment tools are not routinely used, and nurses often rely on their own knowledge to conduct skin assessments and judge patients at risk (Hulsenboom *et al*. 2007, Samuriwo & Dowding 2014). In nursing homes, nurses spend a lot of time on documentation and communication (Munyisia *et al*. 2011b, Dellefield *et al*. 2012); however, incomplete documentation remains an issue (Wang *et al*. 2015), suggesting the need for an increased focus on the accuracy of documentation (Wang *et al*. 2011).

To avoid the consequences of PUs, it is important to gain knowledge about the accuracy of nursing documentation related to PUs and how nurses in nursing homes communicate PU prevention strategies. An audit of record accuracy may provide important information about the documentation of prevalence, risk factors and prevention of PUs. In addition, patient examinations can provide information about the accuracy of the nursing documentation, and what nurses are actually doing and observing for their patients.

### Background

#### PU prevalence, risk factors and prevention

A recent systematic review highlighted that no single factor can explain the risk for PUs (Coleman *et al*. 2013). However, increased age, decreased mobility and acute and chronic illnesses increase a patient's risk for developing PUs (Bours *et al*. 2002, McInnes *et al*. 2011). PUs may cause pain, prolong hospital stays and increase patients' complications as well as social burden. In addition, PUs have an economic cost for patients, institutions and society in general (McInnes *et al*. 2011).

There is a lack of knowledge in nurses working in nursing homes about PU prevention (Demarré *et al*. 2012), with several studies noting a gap between research and practice in PU prevention (Pancorbo‐Hidalgo *et al*. 2006, Chang *et al*. 2010, Meesterberends *et al*. 2011). A recent review found no evidence that implementing standardized PU risk assessment scales had an impact on clinical practice, although there was rationale for using these scales as quality indicators for the care process (Kottner & Balzer 2010).

Another review found limited evidence for PU prevention interventions in adults (Gillespie *et al*. 2014). However, a comparison of support surfaces found that foam alternatives reduced the incidence of PUs among at‐risk patients compared with standard hospital foam mattresses. Studies have also shown sheepskin to be effective in reducing the incidence of PUs (McInnes *et al*. 2015). The European Pressure Ulcer Advisory Panel (EPUAP) and the Pressure Ulcer Advisory Panel (NPUAP) have developed international guidelines based on recent evidence. These guidelines provide evidence‐based healthcare recommendations to prevent the development of PUs.

#### Nursing documentation in nursing homes

Documentation is an important information source when judging the quality of nursing care. However, studies have found major limitations in nursing documentation as a tool for planning and evaluating nursing care in nursing homes (Ehrenberg *et al*. 2001, Wang *et al*. 2015). An increased focus on the accuracy of nursing documentation was recommended in an extensive review conducted by Wang *et al*. (2011).

Two studies in hospital settings that conducted patient examinations using the Braden scale (Bergstrom *et al*. 1987) and the EPUAP form (European Pressure Ulcer Advisory Panel 2009) found differences in the proportion of PUs recorded in an examination compared with the nursing records (Gunningberg 2004, Thoroddsen *et al*. 2013), with the proportion differing by up to 40% (Gunningberg & Ehrenberg 2004, Thoroddsen *et al*. 2013). A Swedish study examined 413 electronic health records (EHRs) and assessed the same patients at a university hospital. Only 14·3% of PUs were documented in the EHRs, compared with 33·3% revealed during skin examinations (Gunningberg & Ehrenberg 2004). Despite the increased focus on the importance of accurate nursing documentation in improving patient outcomes, no comparison between documentation and assessment of nursing home residents has been conducted to date. This study aimed to describe the accuracy and quality of nursing documentation of PU prevalence, risk factors and prevention and compare retrospective audits of nursing documentation with patient examinations conducted in nursing homes.

## The study

### Design

This study used a cross‐sectional, descriptive design and was conducted in five nursing homes from three municipalities in southern Norway throughout January ‐ February 2013.

### Method

**Figure 1:**
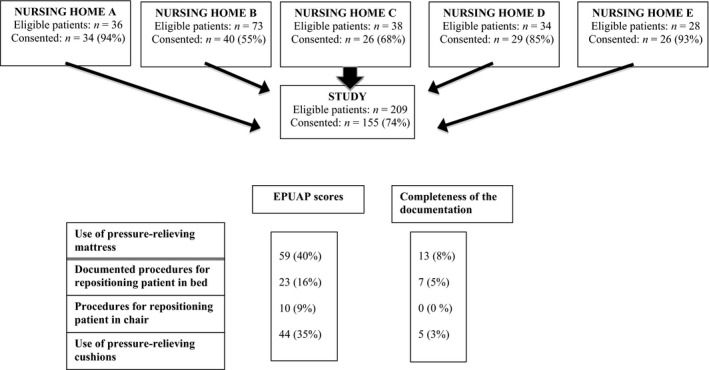
Number of nursing homes and patients included in this study and comparison of the European Pressure Ulcer Advisory Panel (EPUAP) scores with four pressure ulcer prevention interventions documented in patient records.

Nursing homes were recruited through an email sent to nursing home managers in the municipalities connected to the Centre for Caring Research, southern Norway. Managers who wished to participate were invited to contact the project manager, one of the present authors (RLH), by phone or email. The inclusion criterion was all patients currently living in the nursing homes. Ethical considerations lead to the exclusion of terminal patients and those considered by nursing staff to be too unwell. In total, 209 patients were invited to participate, and 155 (74%) patients or their proxies gave informed consent. Four of the five nursing homes had permanent‐stay patients, including 2‐4 patients in residential respite care or short‐term stay (Figure 1). One ward refused to participate.

### Measurements

Three measurement instruments were used: the Braden scale (Bergstrom *et al*. 1987, Braden & Bergstrom 1994), translated into Norwegian (National Pressure Ulcer Advisory Panel, European Pressure Ulcer Advisory Panel and Pan Pacific Pressure Injury Alliance 2014); the EPUAP form for registering PUs, based on guidelines developed by the EPUAP and the NPUAP (Beeckman *et al*. 2007); and an audit instrument developed by Gunningberg and Ehrenberg (2004) and translated into Norwegian by Fossum *et al*.(2011).

The Braden scale covers six different variables: (1) Sensory perception; (2) Degree to which skin is exposed to moisture; (3) Physical activity; (4) Mobility; (5) Food intake, nutrition and (6) Friction and shear. Variables one to five are scored on a scale from 1‐4, while variable six is scored from 1‐3. The scores for all six variables are added to form a summative total score. The Braden scale has been shown to be both valid and reliable (Bergstrom *et al*. 1987, Braden & Bergstrom 1994).

The EPUAP form records age, sex, whether the patient lives in his/her own home or a nursing home, whether the patient is in the nursing home short‐term or permanently, and the patient's height and weight. This form also includes the Braden scale as a separate checklist; however, in this study only data from the separate Braden scale was used because this scale had a more detailed guiding text. The skin inspection details the observed PU categories (grades). The EPUAP form notes the locations of the highest grade PUs (sacrum, heel, hip, other) and all existing PUs, documenting them on an indicator poster. The form also documents whether and what type of preventive measures are used. The EPUAP form showed excellent agreement in tests of inter‐rater reliability (Bours *et al*. 1999, Demarré *et al*. 2012).

The audit instrument (Gunningberg & Ehrenberg 2004) contains 43 variables, including the patient's age, sex, total length of stay in the nursing home (months) and diagnoses (other variables are presented in Tables 3 and 4). Some of these variables require a yes or no answer; if “yes”, follow‐up questions must be answered, such as “Can you see any gradation in the patient's records? If yes, list the order of degree.”

### Data collection

Each nursing home had one or two nurses responsible for data collection. These nurses underwent a 2‐hour education session conducted by one of the present authors (RLH) concerning the forms and grading of PUs. One of the present authors (RLH) had overall responsibility for data collection, which was conducted over 1 week in each nursing home.

After the patients or their proxies had given written consent to participate, patient records were printed and de‐identified, and the patients were assessed with the Braden scale and the EPUAP form. The patient journal information included nursing care plans, medication charts, progress notes and summaries from the last 3 months. In general, the same nurses completed the assessment instrument for all patients in one ward.

Four nursing homes had the same EHR systems and three of these reported all nursing documentation in progress notes. In one nursing home, only nurses completed documentation using the code ‘nursing documentation’, while nurses, technicians and assistants completed documentation under an ‘assistants’ code. One nursing home used a different EHR system and used progress notes instead of nursing care plans. The patient records varied from four pages to more than 90 pages. Nineteen of the 155 patient records were audited by two of the authors (RLH;MF), and consensus was achieved by discussion. The remaining records were audited by one of the authors (RLH). Data from the audit were recorded on the audit instrument and then entered into the SPSS programme, version 19 (IBM SPSS Statistics for Windows, Version 19·0.; IBM Corp., Armonk, NY, USA).

### Data analysis

All analyses were conducted using SPSS, version 19 (IBM SPSS Statistics for Windows, Version 19·0.; IBM Corp.). Most outcome variables were recorded as categorical or ordered categorical data. Thus, frequencies, proportions median (md) and quartiles (Q_1_;Q_3_) were used for statistical description (Altman 1991). Based on this study, the prevalence (p %) of PUs in nursing home patients was estimated by the 95% confidence interval (95% CI) of the proportion patients with PU according to the patient examinations, and compared with corresponding estimation of the prevalence based on the content of the nursing documentation. The discrepancy in the proportion findings of within‐patient differences between the examination and the nursing documentation was evaluated by sets of paired data, and expressed as the paired proportion patients (p%; 95% CI) with missing nursing documentation in relation to the patient examination or vice versa (Altman *et al*. 2000).

The PU level in the patient records was rated as: no ulcer, stage I, stage II, stage III or stage IV ulcers (recoded as 0, 1, 2, 3 and 4). If a patient had several PUs, they were all noted in both the patient examinations and patient records.

In total, 19 (12%) of the patient records were assessed by two raters. Their scores were compared, and the inter‐rater agreement adjusted for chance was calculated with Cohen's kappa. Kappa values between 0·81‐1·00 are regarded as indicating a very good agreement, kappa 0·61‐0·80 as good, 0·41‐0·60 as moderate, 0·21‐0·40 as fair and lower values indicate poor agreement (Altman 1991).

### Ethics

This study was approved by the Regional Committee for Medical Research Ethics in southern Norway (REK sør, reference number 2012‐1642‐REK), and by the Norwegian Social Science Data Services (project number 32123). Patients were informed in writing and verbally about the study. Between 70‐80% of patients in nursing homes suffer from dementia, although many are not diagnosed (The National Directorate for Health and Sosial Affairs 2009). Accordingly, it is difficult to ensure patient autonomy despite written consent requirements. The high number of patients suffering from dementia was a key reason why nurses on the wards completed the patient examinations. The patients' PU risks were documented in their patient records for follow‐up. When patients were not able to give consent to participate, consent was obtained from the person listed as the patient's proxy. The research team was not informed about the number of proxies or spouses who consented on a patient's behalf.

## Results

At baseline, 155 patients participated. Of these, 109 (77%) were aged over 80 years, and 108 (72%) were women. A total of 112 (75%) patients were permanent nursing home residents (Table 1). The agreements between the two raters for all the variables in the audit instrument were between k = 0·58 and 1·00, indicating moderate to very good agreement, with the percentage of agreements between 54% and 100%.

**Table 1:** Patient characteristics (*n *=* *155)

Characteristics	Frequency *n* (%)
Age (*n *=* *141)	
40‐59 years	1 (0)
60‐69 years	5 (4)
70‐79 years	26 (18)
80‐89 years	69 (49)
>89 years	40 (28)
Sex (*n *=* *151)	
Female	108 (72)
Male	43 (29)
Type of ward (*n *=* *150)	
Residential respite care	38 (25)
Permanent stay	112 (75)

### Paired comparisons of the record contents and patient examinations

The comparisons between the 155 patient examinations and the content of the nursing documentation showed that the prevalence of PUs was 33 (21%, 95% CI, 21‐29%) according to the patient examinations. Correspondingly, the prevalence of PUs according to the content of the nursing documentations was slightly different, 38 (25%; 95% CI, 19‐32%), as indicated by the two overlapping confidence intervals. However, according to the paired comparisons of patient examinations and the nursing documentations, only 18 (54·5%) patients with assessed PU in the examinations were also found in the content of the nursing documentations. The remaining 20 patients with documented PU were assessed with ‘no PU’ in the examinations, and another 15 patients (45·5%. 95% CI, 30‐62%) with assessed PU in the examinations were not documented as having PU in their patient records. This indicates that based on this study one can expect lack of reporting in 30‐62% of patients with PUs. Furthermore, 17 (45%) of the documented 38 PUs were not graded.

When comparing complete pairs of data from patient examinations and the corresponding contents of the patient records, 59 (40%) of 146 patients received pressure‐relieving mattress prevention and 10 of these patients had pressure‐relieving mattress documented in their patient records. This result shows that 49 (83%, 95% CI, 72‐91%) of the pressure‐relieving mattresses were not documented. Correspondingly, regarding the prevention of PUs in chairs, 44 (35%, 95% CI 27‐44%) of the 126 patients of complete pairs of data had pressure‐relief cushions in the chair, but 42 (96%, 95% CI, 86‐99%) of these patients did not have pressure‐relief cushions in the chair documented in their patient records.

Procedures of repositioning in bed were assessed and identified in 23 (16%) of 140 patient examinations. Eighteen of these identified patients (78%, 95% CI, 58‐90%) did not have procedures of repositioning in bed documented. Correspondingly, procedures of repositioning in chair were assessed and identified in 10 (16%) of 118 patient examinations. About ten (100%, 95% CI, 72‐100%) patients did not have the repositioning in chair documented.

### Patient EPUAP and Braden Scale Evaluations

Braden scores from patient examinations were reported for 149‐153 patients; although 155 patients consented to participate, six patients had incomplete data. Table 2 shows that the median and quartile scores for the six Braden scale items differed slightly.

**Table 2:** Braden scores, pressure ulcer prevalence and interventions assessed in nursing home patients (*n *=* *155)

Characteristics	
Braden score: number of residents (a *n *= 153)	
Sensory perception (*n *=* *149): md^b^ (Q1;Q3)^c^	3 (3;4)
Degree to which skin is exposed to moisture (*n *=* *152): md (Q1;Q3)	4 (3;4)
Physical activity (*n *=* *150): md (Q1;Q3)	3 (2;4)
Mobility (*n *=* *153): md (Q1;Q3)	3 (2;4)
Food intake, nutrition (*n *=* *153): md (Q1;Q3)	3 (3;4)
Friction and shear (*n *=* *149): md (Q1;Q3)	2 (2;3)
Braden score total (*n *=* *149): md (Q1;Q3)	18 (16;18)
Prevalence of pressure ulcers (a *n *=* *154)	
No pressure ulcer: *n* (%)	121 (79)
Stage 1: *n* (%)	20 (13)
Stage 2: *n* (%)	6 (4)
Stage 3: *n* (%)	4 (3)
Stage 4: *n* (%)	3 (2)
Prevention of pressure ulcers in bed (*n *=* *146)	
No pressure‐relieving mattress: *n* (%)	87 (60)
Pressure‐relieving mattress with or without motor: *n* (%)	59 (40)
Pressure‐relieving pillow for heels in bed (*n *=* *92)	
Yes: *n* (%)	32 (35)
No: *n* (%)	60 (65)
Prevention of pressure ulcers in a chair (*n *=* *126)	
No pressure‐relieving cushion: *n* (%)	82 (65)
Pressure‐relieving pillow without motor: *n* (%)	44 (35)
Repositioning of the patient in bed (*n *=* *140)	
Yes: *n* (%)	23 (16)
No: *n* (%)	117 (84)
Repositioning of the patient in chair (*n *=* *118)	
Yes: *n* (%)	10 (9)
No: *n* (%)	108 (91)

a Missing data.

b Median (md).

c Inter‐quartiles range (Q1;Q3).

The number of PUs was 33 (22%), categorized into four stages: stage 1 = 20 (13%), stage 2 = 6 (4%), stage 3 = 4 (3%) and stage 4 = 3 (2%). In total, 59 patients (40%) had a pressure‐relieving mattress with or without a motor, and 32 patients (35%) had pressure‐relieving pillows on their beds. Forty‐four patients (35%) had pressure‐relieving cushions in their chairs; 23 (16%) had procedures for repositioning in bed and 10 (9%) chair‐bound patients had repositioning procedures.

### Prevalence, Risk Factors and Prevention of PUs in Nursing Documentation

Table 3 shows the PUs documented in the patient records. Thirty‐eight patients (26%) had PUs recorded in the nursing documentation, categorized as: stage 1 = 9 (6%), stage 2 = 10 (7%), stage 3 = 1(1%) and stage 4 = 1(1%). The remaining 17 (11%) patients had an undocumented PU level. Pressure‐relieving mattresses with or without a motor were noted in nursing documentation for 13 patients (8%). Seven patients (5%) had pressure‐relieving cushions in their chairs; seven (5%) patient records documented procedures for repositioning patients in bed and zero (0%) documented repositioning procedures for a chair‐bound patient.

**Table 3:** Nursing documentation for risk and prevalence of pressure ulcers: completeness (*n *=* *155)

Variables	*n* (%)
Prevalence of pressure ulcers in patient records	
No pressure ulcer	117 (76)
Stage 1	9 (6)
Stage 2	10 (7)
Stage 3	1 (1)
Stage 4	1 (1)
Undocumented degree of pressure ulcer	17 (11)
Prevention of pressure ulcers in bed	
No pressure‐relieving mattress	142 (92)
Pressure‐relieving mattress with or without motor	13 (8)
Prevention of pressure ulcers in a chair	
No pressure‐relieving cushion	150 (97)
Pressure‐relieving pillow with or without motor	5 (3)
Procedures for repositioning the patient in bed	
Yes	7 (5)
No	148 (96)
Procedures for repositioning the patient in chair	
Yes	0 (0)
No	155 (100)

Table 4 presents the completeness of the nursing documentation in terms of PU risk and prevalence. In 116 patient records (75%), patient discomfort or the need to change positions was described. The degree of sensory perception was described in terms of three of the following four variables: complete deterioration, *n* = 0 (0%); significantly impaired, *n* = 5 (4%); somewhat weaker, *n* = 15 (9%) and no impairment *n* = 39 (34%).

**Table 4:** Nursing documentation for the assessment and prevention of pressure ulcers: completeness

Documentation in the patient records (*n *=* *155)	*n* (%)
Sensory perception	
(Description of discomfort or the need to change position) If yes	116 (75)
Specified degree of sensory perception	
Complete deterioration	0 (0)
Significantly impaired	5 (4)
Somewhat weaker	15 (9)
No impairment	39 (34)
Degree to which skin is exposed to moisture	
If yes	109 (70)
Degree of moisture	
Constantly moist	0 (0)
Often moist	10 (9)
Somewhat damp	26 (24)
Dry or normal moisture	27 (25)
Physical activity	
If yes	148 (96)
Level of physical activity	
Bedridden	2 (1)
In a wheelchair	31 (21)
Walks with assistance	42 (28)
Walks with and without aids	56 (38)
Mobility	
If yes	148 (96)
Specified degree of mobility	
Bedridden	2 (1)
Very limited	28 (19)
Slightly limited	61 (41)
Unlimited	18 (12)
Food intake, nutrition	
If yes	119 (77)
Specified level of food intake	
Less than half the normal portion	2 (2)
Half of the normal portion	1 (1)
Three‐fourths of the normal portion	2 (2)
Normal portion	8 (7)
Friction and shear	
If yes	9 (6)
Grade not specified	9 (100)

## Discussion

Our study highlights a gap between the use of preventive strategies documented in patient records and data from patient examinations in nursing homes. Lack of accuracy may challenge the use of patient records as a valid source of information in nursing practice. Our results are similar to findings from a study conducted by Gunningberg and Ehrenberg (2004) in a hospital setting, and other studies conducted in aged care facilities (Schnelle *et al*. 2004, Fossum *et al*. 2013, Alexander 2015). The results of this study were derived from an audit of patient records. As nurses in nursing homes frequently use other sources of information, such as oral handover between shifts, a strong tradition of oral communication in nursing may have influenced our results. However, the PU prevalence rate was consistent with those reported in other international studies, but slightly lower for severe PU stages (Vanderwee *et al*. 2007, Moore & Cowman 2012, Bååth *et al*. 2014). Although several prevention strategies are commonly implemented in healthcare services, the PU prevalence appears to be at the same level (Bååth *et al*. 2014).

Our results of paired comparisons showed differences between the prevention strategies documented in the patient records and assessed prevention strategies, such as the use of pressure‐relieving mattress, repositioning the patient in bed or in a chair and the use of pressure‐relieving cushions. Based on the results of this study, it is likely that a lack of recording of procedures of repositioning the patient in bed may be identified in 58‐90% of patient records, and a lack of documented pressure‐relieving mattresses may be identified in 72‐91% of patient records. Patients at high risk for developing PUs should use pressure‐relieving mattresses instead of standard hospital foam mattresses (McInnes *et al*. 2011), and alternating pressure mattresses may be more cost‐effective than alternating pressure overlay mattresses (McInnes *et al*. 2011). An earlier study conducted in a hospital setting showed similar results to our study, with nurses performing more interventions than they recorded in patient records (Gunningberg & Ehrenberg 2004). However, this earlier study also showed similar differences in the number and grades of PUs between the record audits and the patient examinations. These results differed from our results, where nurses documented more pressure injuries but the documentation lacked accuracy and was incomplete. A reason for the discrepancy between the records and examinations may be that nurses in nursing homes do not have time, skills and knowledge to update patient records. A study conducted in nursing homes has shown that nurses and nursing assistants in nursing homes have a lack of knowledge about PU prevention (Demarré *et al*. 2012), and continuing PU prevention education and use of PU ‘champions’ may improve the accuracy and quality of nursing documentation (Sullivan & Schoelles 2013).

Patients in nursing homes are commonly aged over 80 years and have a variety of additional diseases, making prevention measures important (The National Directorate for Health and Sosial Affairs 2009). The results of our study showed that nurses undertake more PU prevention than they document in patient records. Underreporting of PU prevention efforts may be of concern for nursing home managers in terms of competence (McInnes *et al*. 2011) and economics (Bennett *et al*. 2004, Whittington *et al*. 2004, McInnes *et al*. 2011). A previous study concluded that documentation did not reflect the use of systematic assessment and research‐based instruments to determine whether patients had PUs or were at risk for developing PUs (Gunningberg *et al*. 2001); findings consistent with the results of our study.

Despite an increased focus on PU prevention, the lack of accuracy in nursing documentation should be addressed. Implementing EHRs with decision support tools may be one way to address this issue and improve the quality and accuracy of documentation in nursing homes (Munyisia *et al*. 2011a, 2012, Wang *et al*. 2013).

### Methodological limitations

Owing to ethical issues, several nurses completed the data collection rather than one person, which may have had an impact on the reliability of data collected. However, one of the present researchers was in attendance at the nursing homes during data collection to avoid errors in collected data. As PUs are associated with poor care, underreporting of PUs might have occurred; however, the prevalence of PUs was similar to other studies from nursing homes (Vanderwee *et al*. 2007, Fossum *et al*. 2011), and the nurses that completed the data collection received instruction and education to develop their data collection techniques.

The validity of the results of this study may be limited because of the exclusion criteria (terminal patients and those unwell to participate). Patients unable to consent and with spouses/proxies that were difficult to contact were not included. As other relevant characteristics such as diagnosis were not collected, non‐participating patients may have had worse health conditions than the participants. Overall, the agreement between the two raters was moderate to very good, and our results were consistent with other studies.

## Conclusions

There is a gap between nursing practice and nursing documentation in nursing homes. Nurses may need training and education to perform high quality PU prevention and complete accurate nursing documentation for patients in nursing homes. We found inaccuracies in the nursing documentation in nursing homes, indicating that it is necessary to focus on organizing clinical practice to ensure nurses have the opportunity to use available guidelines and document their nursing practice. Further research should explore different EHRs systems and identify standardization that may support nurses to perform more complete and accurate documentation of their practice in nursing homes.

## Conflict of interest

No conflict of interest.

## Author contributions

Study design: RL.H, M.F; data collection: RL.H, M.F; analysis and interpretation of the results: RL.H, M.F; manuscript preparation: RL.H, M.F.

All authors have agreed on the final version and meet at least one of the following criteria [recommended by the ICMJE (http://www.icmje.org/recommendations/)]:

- substantial contributions to conception and design, acquisition of data, or analysis and interpretation of data;
- drafting the article or revising it critically for important intellectual content.
